# Computed Tomographic Radiomics in Differentiating Histologic Subtypes of Epithelial Ovarian Carcinoma

**DOI:** 10.1001/jamanetworkopen.2022.45141

**Published:** 2022-12-05

**Authors:** Mandi Wang, Jose A. U. Perucho, Yangling Hu, Moon Hyung Choi, Lujun Han, Esther M. F. Wong, Grace Ho, Xiaoling Zhang, Philip Ip, Elaine Y. P. Lee

**Affiliations:** 1 Department of Radiology, Shenzhen People's Hospital (The Second Clinical Medical College, Jinan University; The First Affiliated Hospital, Southern University of Science and Technology), Shenzhen, Guangdong, China; 2 Department of Diagnostic Radiology, School of Clinical Medicine, The University of Hong Kong, Hong Kong Special Administrative Region, China; 3 Department of Radiology, Heersink School of Medicine, The University of Alabama at Birmingham, Birmingham; 4 Department of Radiology, The First Affiliated Hospital, Sun Yat-sen University, Guangzhou, China; 5 Department of Radiology, Eunpyeong St. Mary’s Hospital, College of Medicine, The Catholic University of Korea, Seoul, Korea; 6 Department of Radiology, Sun Yat-sen University Cancer Center, Guangzhou, China; 7 Department of Radiology, Pamela Youde Nethersole Eastern Hospital, Hong Kong Special Administrative Region, China; 8 Department of Radiology, Queen Mary Hospital, Hong Kong Special Administrative Region, China; 9 Department of Pathology, Queen Mary Hospital, School of Clinical Medicine, The University of Hong Kong, Hong Kong Special Administrative Region, China

## Abstract

**Importance:**

Epithelial ovarian carcinoma is heterogeneous and classified according to the World Health Organization Tumour Classification, which is based on histologic features and molecular alterations. Preoperative prediction of the histologic subtypes could aid in clinical management and disease prognostication.

**Objective:**

To assess the value of radiomics based on contrast-enhanced computed tomography (CT) in differentiating histologic subtypes of epithelial ovarian carcinoma in multicenter data sets.

**Design, Setting, and Participants:**

In this diagnostic study, 665 patients with histologically confirmed epithelial ovarian carcinoma were retrospectively recruited from 4 centers (Hong Kong, Guangdong Province of China, and Seoul, South Korea) between January 1, 2012, and February 28, 2022. The patients were randomly divided into a training cohort (n = 532) and a testing cohort (n = 133) with a ratio of 8:2. This process was repeated 100 times. Tumor segmentation was manually delineated on each section of contrast-enhanced CT images to encompass the entire tumor. The Mann-Whitney *U* test and voted least absolute shrinkage and selection operator were performed for feature reduction and selection. Selected features were used to build the logistic regression model for differentiating high-grade serous carcinoma and non–high-grade serous carcinoma.

**Exposures:**

Contrast-enhanced CT-based radiomics.

**Main Outcomes and Measures:**

Intraobserver and interobserver reproducibility of tumor segmentation were measured by Dice similarity coefficients. The diagnostic efficiency of the model was assessed by receiver operating characteristic curve and area under the curve.

**Results:**

In this study, 665 female patients (mean [SD] age, 53.6 [10.9] years) with epithelial ovarian carcinoma were enrolled and analyzed. The Dice similarity coefficients of intraobserver and interobserver were all greater than 0.80. Twenty radiomic features were selected for modeling. The areas under the curve of the logistic regression model in differentiating high-grade serous carcinoma and non–high-grade serous carcinoma were 0.837 (95% CI, 0.835-0.838) for the training cohort and 0.836 (95% CI, 0.833-0.840) for the testing cohort.

**Conclusions and Relevance:**

In this diagnostic study, radiomic features extracted from contrast-enhanced CT were useful in the classification of histologic subtypes in epithelial ovarian carcinoma. Intraobserver and interobserver reproducibility of tumor segmentation was excellent. The proposed logistic regression model offered excellent discriminative ability among histologic subtypes.

## Key Points

**Question:**

Are computed tomographic radiomics clinically valuable in differentiating histologic subtypes of epithelial ovarian carcinoma?

**Findings:**

In this diagnostic study of 665 patients with epithelial ovarian carcinoma, a logistic regression model developed by using selected radiomic features achieved excellent performance for the classification of high-grade serous carcinoma and non–high-grade serous carcinoma, with areas under the curve of 0.837 for the training cohort and 0.836 for the testing cohort.

**Meaning:**

The proposed model based on contrast-enhanced computed tomographic radiomics was useful in differentiating histologic subtypes of epithelial ovarian carcinoma and therefore could benefit clinical management and prognosis evaluation.

## Teaser

This diagnostic study of women with epithelial ovarian carcinoma assesses whether computed tomography–based radiomic features can differentiate histologic subtypes of epithelial ovarian carcinoma.

## Introduction

Epithelial ovarian carcinoma (EOC) is the leading cause of death among women with gynecologic malignant tumors.^1^ Most EOCs are typically detected at advanced stages, usually with peritoneal metastases. Despite the use of new chemotherapy regimens and targeted therapies, the prognosis remains unsatisfactory, with a 5-year survival rate less than 45%.^2^

Histologic subtypes and International Federation of Gynecology and Obstetrics (FIGO) stages are crucial characteristics for treatment stratification as well as disease prognostication. Epithelial ovarian carcinoma can be classified as high-grade serous carcinoma (HGSC) and non-HGSC according to the different pathways of carcinogenesis.^3^ High-grade serous carcinoma is the most frequent and lethal subtype, accounting for 70% of EOC.^4^ Non-HGSC consists of low-grade serous carcinoma, mucinous carcinoma, endometrioid carcinoma, clear cell carcinoma, and malignant Brenner tumors. Non-HGSC presents as an indolent behavior and progresses through a stepwise mutation process, whereas HGSC tends to be more aggressive with greater genetic instability and thereby metastasizes rapidly.^5,6^ With better understanding of the molecular events that support ovarian carcinogenesis, newer tailored therapies and subtype-specific research could be investigated.

For the treatment of FIGO stage III or IV EOCs, which are mostly HGSCs, neoadjuvant chemotherapy may be considered when complete debulking is unlikely to be achievable with primary cytoreductive surgery.^7^ However, for non-HGSC, such as low-grade serous carcinoma, clear cell carcinoma, and mucinous carcinoma, primary cytoreductive surgery is recommended because of its resistance to conventional taxane or platinum chemotherapy.^8^ Aside from treatment stratification, genomic instability in HGSC can be a target for therapeutic agents. Therefore, accurate identification of histologic subtypes is vital and will be beneficial for personalized management. In clinical practice, histologic diagnosis is made through surgery or tissue biopsy. Intraoperative frozen section could assist in histologic classification but could still result in misdiagnosis, not to mention the invasive nature of these procedures and associated increase in intraoperative time.^9^

Malignant tumors are heterogeneous with intratumor spatial variations at both morphologic and histopathologic levels, such as cellularity, angiogenesis, necrosis, and extravascular extracellular matrix.^10^ Epithelial ovarian carcinoma has intratumor heterogeneity, especially in large ovarian masses. Radiomics analysis is emerging as a noninvasive and useful tool to assess highly heterogenous malignant tumors, such as HGSCs.^11^ Radiomics is a mathematical quantitative analysis that converts medical images into minable, high-dimensional data by extracting large amounts of mathematical features.^12^ Previous studies reported the ability of computed tomography (CT)–based radiomics in the evaluation of EOC, including differentiating between EOC and non-EOC, as well as predicting clinical outcome or survival.^13,14,15^ Recent studies showed promising results of CT-based texture features in classifying HGSC and non-HGSC^16^ and excellent performance of multiple parametric magnetic resonance imaging (MRI)–based radiomics in distinguishing type 1 (non-HGSC) and type 2 (HGSC) tumors.^17,18,19^ Nonetheless, to date, none of the studies focused on the discriminative ability of CT radiomics in histologic subtyping using multicenter data sets. Multicenter radiomics analysis is crucial in advancing the translation and integration of radiomics in clinical management. To validate these findings based on a large cohort across multiple centers in East Asia, with different machines and varied imaging parameters, the aim of this multicenter study was to assess the value of CT-based radiomic features in histologic subtyping of EOC.

## Methods

This multicenter retrospective study was approved by the relevant local institutional review boards and performed in accordance with the Helsinki Declaration.^20^ Informed consent was waived by the local institutional review boards because this study involved anonymized human data without identifying information that had already been collected. The study included developing, training, and testing a prediction model for diagnostic purpose, and it followed the Transparent Reporting of a Multivariable Prediction Model for Individual Prognosis or Diagnosis (TRIPOD) reporting guideline.

### Study Patients

**Figure 1.**
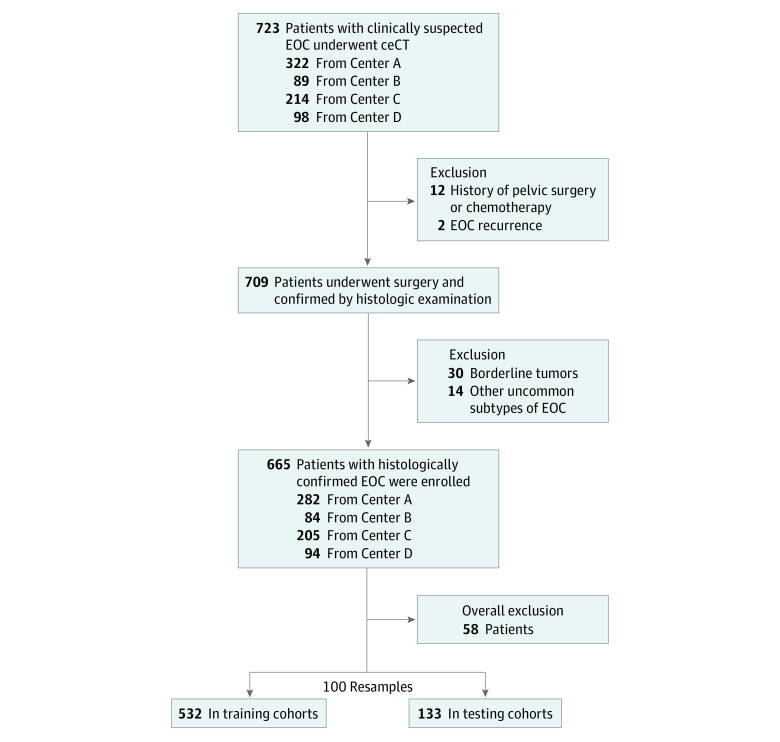
Diagram of Patient Inclusion ceCT indicates contrast-enhanced computed tomography; EOC, epithelial ovarian carcinoma.

Potential patients with EOC for this multicenter, retrospective diagnostic study were drawn from databases from local hospitals and collaborating institutes in Hong Kong, Guangdong Province of China, and Seoul, South Korea, between January 1, 2012, and February 28, 2022. Inclusion criteria included patients (1) with histologically confirmed HGSC or non-HGSC of EOC, (2) without prior history of pelvic surgery or treatment, and (3) who underwent pretreatment contrast-enhanced CT (ceCT). Patients with EOC recurrence and incomplete clinicopathological records were excluded. The diagram of patient inclusion is displayed in Figure 1.

All patients underwent hysterectomy and bilateral salpingo-oophorectomies with or without debulking operations. All surgical and biopsy specimens were reviewed by board-certified pathologists (including P.I.) from the respective centers, and histologic subtypes were recorded. Histologic subtypes were dichotomized to HGSC and non-HGSC EOC.

### Imaging Acquisition

Abdominopelvic ceCT scans were acquired for all patients preoperatively. These procedures were performed on multiple scanners in the 4 participating centers. The CT scanners and imaging parameters are tabulated in the eTable in the Supplement.

### Tumor Segmentation

All ceCT images were 3σ normalized and reconstructed to 5-mm section thickness for segmentation and radiomics analysis. Tumor segmentation was manually delineated using 3D Slicer version 4.11^21^ or ITK-SNAP version 3.6.0.^22^

Tumor segmentation was delineated by different radiologists from multiple centers. For the cases from centers A and B, the first radiologist (R1, 3 years’ experience in pelvic imaging) (M.W.) delineated the tumors on ceCT images. Tumor segmentation was strictly delineated around the border of the tumor on each section containing the tumor. Subsequently, all the segmentations were verified by a second radiologist (R2, >10 years’ experience in pelvic imaging) (E.Y.P.L.). In cases of disagreement, the final segmentation was determined by consensus. For the cases in centers C and D, a third radiologist (R3, 2 years’ experience in radiology) (Y.H.) and a fourth radiologist (R4, >10 years’ experience in radiology) (M.H.C.) contoured the tumors independently. If bilateral pelvic masses were detected, only the larger mass was delineated. All the radiologists were blinded to the clinicopathological results.

### Intraobserver and Interobserver Reproducibility Evaluation of Tumor Segmentations

Fifty cases of ceCT images from center A were randomly selected for the evaluation of intraobserver and interobserver reproducibility in tumor segmentations. To evaluate the intraobserver agreement, R1 (who delineated the segmentation for centers A and B) and R4 (from center D) repeated the delineation of the 50 cases twice with an interval of more than 1 month. To evaluate the interobserver agreement, tumor segmentation of the 50 cases were drawn by R1, R3, and R4, independently. Dice similarity coefficients (DSCs) were calculated for evaluating intraobserver and interobserver agreement. A DSC greater than 0.80 was considered as satisfactory reproducibility.

### Feature Extraction

Radiomics features were extracted using the open-source package PyRadiomics, version 3.0.^23^ Bin width was set to 25. Seven classes of radiomic features were extracted from ceCT images: shape, first order, gray-level co-occurrence matrix, gray-level size zone matrix, gray-level run length matrix, gray-level dependence matrix, and neighboring gray tone difference matrix features. In addition to these original features, Laplacian of Gaussian features with σ of 1 to 5 and wavelet features with transformed images that yielded 8 decompositions were also extracted. In total, 1288 radiomics features were extracted for each patient.

### Statistical Analysis

#### Feature Reduction and Selection

The recruited patients were randomly divided into training and testing cohorts with a ratio of 8:2 using stratified sampling according to the distribution of histologic subtypes. This procedure was repeated 100 times to produce 100 resamples of the 8:2 split.

For each resample, the following procedures were performed. First, the Mann-Whitney *U* test was used to compare the differences of radiomics features between HGSC and non-HGSC. Only significant features were considered for the next step. A 2-tailed *P* < .05 was considered statistically significant. Second, least absolute shrinkage and selection operator (LASSO) regression was used to select useful features in each resample. Third, once steps 1 and 2 were repeated for all resamples, the features were selected and their LASSO coefficients in each resample were noted and tallied, and features were then ranked (in descending order) by the number of times they were selected in a particular resample. Fourth, the top 10% of features (total of 20) or the features that were the most frequently selected in the individual resamples were considered for final model building. Fifth, for each selected feature, their LASSO coefficients across all resamples were averaged. Sixth, the radscore of each patient was then calculated by multiplying each feature’s value by its averaged LASSO coefficient determined in step 6 and then summing those 20 products.

**Figure 2.**
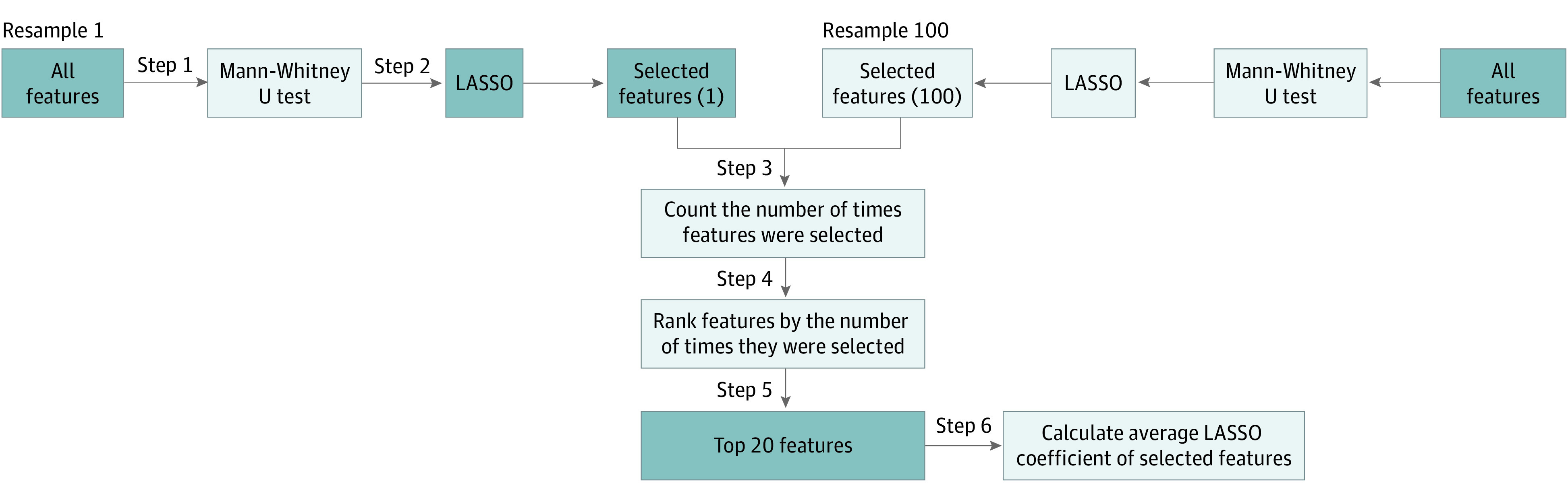
Workflow Diagram of the Voted Least Absolute Shrinkage and Selection Operator (LASSO) Method

LASSO is an L1 regularization–based generalized linear regression analysis method that was widely used as an automatic variable selection tool in radiomics.^24^ The method aims to identify variables that lead to a model that minimizes prediction error. However, it was noted that LASSO regression chose slightly different features across the various resamples. This “voted LASSO” method, described previously, uses each resample’s LASSO results to vote for the most useful features to minimize cohort selection bias in the feature selection process. A flowchart of this method (steps 1-6 from the procedure outlined in the previous paragraph) can also be found in Figure 2.

#### Logistic Regression

Logistic regression (LR) models with these radscores were built for the classification of histologic subtypes. Receiver operating characteristic analysis was conducted on the LR model to compute the area under the curve (AUC), accuracy, sensitivity, and specificity using 10-fold cross-validation. An AUC of 0.6 to 0.7 was considered moderate, 0.7 to 0.8 was considered acceptable, 0.8 to 0.9 was considered excellent, and greater than 0.9 was considered outstanding accuracy.^25^

All the statistical analyses were calculated using in-house R scripts, version 3.6.2 (R Core Team). A workflow diagram of the radiomics analysis and modeling is presented in the eFigure in the Supplement.

## Results

### Demographic Characteristics

A total of 665 women with newly diagnosed EOC (mean [SD] age, 53.6 [10.9] years; age range, 18-90 years) were recruited from 4 centers. The demographic characteristics and histologic subtypes of the patients are given in the Table.

**Table.** Demographic Characteristics and Histologic Subtypes of Patients With Epithelial Ovarian Carcinoma^a^

Characteristic	Total	Center A	Center B	Center C	Center D
Patients	665 (100)	282 (42.4)	84 (12.6)	205 (30.8)	94 (14.1)
Age, mean (SD), y	53.6 (10.9)	52.8 (9.7)	52.1 (12.5)	54.2 (11.7)	55.9 (10.9)
HGSC	436 (65.6)	213 (32.0)	26 (3.9)	136 (20.5)	61 (9.2)
Non-HGSC	229 (34.4)	69 (10.4)	58 (8.7)	69 (10.4)	33 (5.0)
CCC	98 (14.7)	27 (4.1)	25 (3.8)	31 (4.7)	15 (2.3)
MC	51 (7.7)	20 (3.0)	12 (1.8)	14 (2.1)	5 (0.8)
LGSC	22 (3.3)	13 (2.0)	1 (0.2)	5 (0.8)	3 (0.5)
EC	58 (8.7)	9 (1.4)	20 (3.0)	19 (2.9)	10 (1.5)

Abbreviations: CCC, clear cell carcinoma; EC, endometrioid carcinoma; HGSC, high-grade serous carcinoma; LGSC, low-grade serous carcinoma; MC, mucinous carcinoma.

^a^ Data are presented as number (percentage) of patients unless otherwise indicated.

### Intraobserver and Interobserver Reproducibility of Tumor Segmentations

Among the 50 randomly selected cases from center A, the mean (SD) DSC intraobserver metrics were 0.886 (0.104) for R1 and 0.862 (0.101) for R4. The DSC interobserver metrics were 0.801 (0.140) for R1 (3 years’ experience in pelvic imaging) vs R3 (2 years’ experience in radiology) and 0.823 (0.157) for R1 vs R4 (>10 years’ experience in radiology). All the DSC metrics were larger than 0.80, which showed excellent consistency in both intraobserver and interobserver reproducibility.

### Selected Radiomic Features and Performance of LR Model

**Figure 3.**
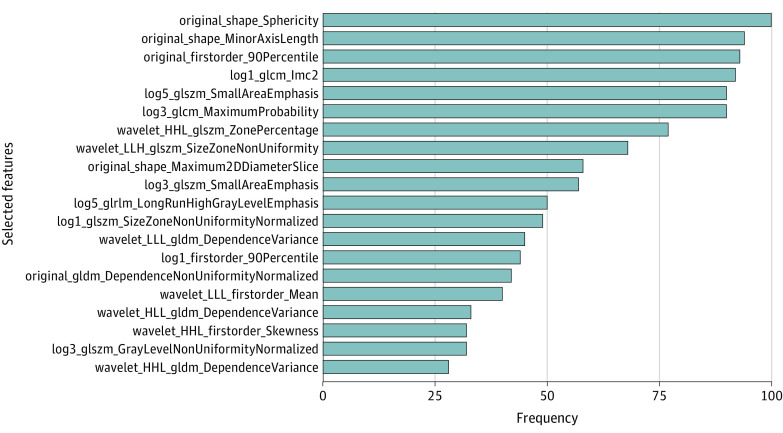
Top 10% of Radiomic Features Selected by Voted Least Absolute Shrinkage and Selection Operator Method Frequency denotes the number of resamples that selected that particular feature. Descriptive definition of each of the selected radiomics features can be found in the eAppendix in the Supplement.

The selected radiomic features using the voted LASSO method and the number of resamples they were selected in are displayed in Figure 3. Sphericity was the top 1 radiomic feature, which was selected 100 times of 100 resamples. The values of sphericity were significantly lower in HGSC than non-HGSC in the whole data set (mean [SD], 0.608 [0.082] vs 0.669 [0.066]; *P* < .001). The descriptive definitions of the selected features can be found in the eAppendix in the Supplement.

**Figure 4.**
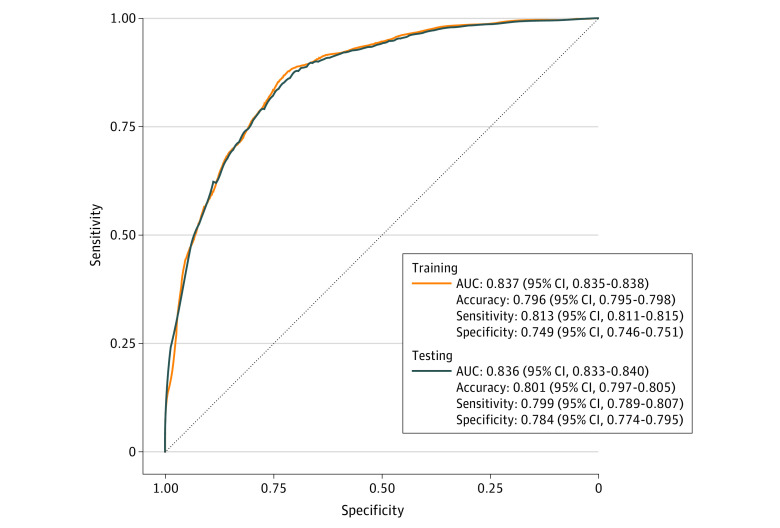
Receiver Operating Characteristic Curves and Metrics for Training and Testing Cohorts of Logistic Regression Model AUC indicates area under the curve.

The AUCs of LR models for differentiating HGSC and non-HGSC were 0.837 (95% CI, 0.835-0.838) for the training cohort and 0.836 (95% CI, 0.833-0.840) for the testing cohort. The receiver operating characteristic curves of training and testing cohorts with performance metrics, including AUC, accuracy, sensitivity, and specificity, are presented in Figure 4.

## Discussion

This multicenter diagnostic study investigated the clinical utility of radiomic features extracted from ceCT based on an LR model in differentiating histologic subtypes of EOC. The proposed CT radiomics model exhibited excellent performance in both the training and testing cohorts, with AUCs of 0.837 and 0.836, respectively.

Histologic subtypes of EOC were classified as HGSC and non-HGSC in our study, according to the different pathways of ovarian tumorigenesis. High-grade serous carcinoma tends to have a poorer prognosis compared with non-HGSC, with an increased risk of death.^26^ Histologic subtyping also helps in treatment stratification and subtype-specific research in EOC. Therefore, the preoperative prediction of the histologic subtypes in EOC could notably benefit the clinical management and prognosis evaluation.

Multicenter data collection is complex, with ethical and regulatory hurdles in sharing imaging data that limit multicenter studies of radiomics research. By considering study effectiveness and labor input, in our study design, we selected a subpopulation of the cohort as representative to test for variability of radiologists of varied experience to measure consistency in tumor delineations, similar to a previous multicenter study.^27^ The intraobserver and interobserver DSC metrics showed excellent reproducibility, suggesting that tumor segmentations from different radiologists were consistent and reliable.

Various radiomic features from different classes were selected for modeling, including original and transformed features from the 6 classes of shape, first order, gray-level co-occurrence matrix, gray-level size zone matrix, gray-level run length matrix, and gray-level dependence matrix features. Among those features, sphericity from the shape class was the top feature, with the highest frequency of 100 in 100 resamples. Similarly, in an MRI radiomics study, sphericity was the optimal selected feature for distinguishing type 1 (non-HGSC) and type 2 (HGSC) EOC among multiple sequences.^18^ Sphericity is a measure of the roundness of the shape relative to sphere, ranging from 0 to 1, with 1 representing a perfect sphere.^28^ We observed that sphericity was significantly lower in HGSC compared with non-HGSC cases. This finding could be associated with the different growth patterns. Non-HGSC presents as an indolent behavior and typically is confined to the ovary. In contrast, HGSC tends to be highly aggressive and genetically unstable and thereby results in an irregular growth pattern and is less likely to be spherical.^5^

A previous study^29^ found that the original features were associated with intratumor heterogeneity based on frequency distribution and spatial complexity of the gray level. In terms of transformed features, plenty of LoG and wavelet features were included in the modeling, which was consistent with the selected features in the MRI radiomics study by Jian et al.^18^ The LoG transform is a convolution kernel filter for edge enhancement that is exploited to smooth the high frequency noise and enhance the variations within the adjacent pixels.^30^ The LoG features present the areas of gray-level change where the σ is defined as the coarseness degree of the emphasized texture. Wavelet transform allows quantification of a high-dimensional, multifrequency texture pattern by yielding 8 decompositions, which are the combinations of a high- or low-pass filter along each of the 3 dimensions.^29,31^ Despite limited clinical relevance on the visual interpretation of the resultant transformed features, these features are considerable components in radiomics and might reveal potential application prospects.^32^

In recent years, a number of CT radiomics studies were conducted to predict the clinicopathological characteristics of ovarian tumors, including discriminating malignant from borderline or benign ovarian masses, differentiating primary and secondary EOC, as well as predicting nodal or abdominopelvic metastases in ovarian cancer.^33,34,35,36,37^ Histologic subtype is an important tumor characteristic that affects clinical management of EOC. Excellent performance of CT radiomics was found in our multicenter study. Relatively high AUCs were achieved in training and testing cohorts, which demonstrated that the proposed LR model using the selected radiomic features provided favorable and robust discriminative ability. Moreover, the sensitivities and specificities of the model were balanced in training and testing cohorts that signified the generalizability of the model. To date, a number of MRI radiomics studies focused on differentiating the histopathological subtypes of EOC.^17,18,19^ The performance of radiomic features extracted from combined MRI sequences were excellent, with AUCs of 0.899 in the training set and 0.806 in the internal validation set.^18^ Although both MRI and CT radiomics offer similarly high AUCs in histologic subtyping, CT is an imaging modality that has wider accessibility and is recommended for initial disease evaluation for treatment stratification and surgical planning in EOC. Moreover, previous MRI radiomics studies were based on data from a single unit, whereas the current study included more patients in training and testing cohorts and was conducted using data from different scanners across multiple centers. Hence, our results may offer better transferability, especially in units where access to MRI is limited.

Although the potential value of radiomics analysis for precision diagnosis, differentiation of characteristics, and prediction of clinical outcome or treatment response have been largely reported in the literature, several challenges and translational gap between ground truth and clinical utility should be considered.^38,39^ To date, there is a lack of standardization in the process of radiomics analysis; thus, the power and reproducibility of the results vary across different methods and models. The proposed workflow in our study tried to provide a practical approach to assess the performance of the prediction model across multicenter data sets. In addition, several comprehensive analyses of various radiomics studies were performed, and a radiomics quality score was introduced to appraise the methodologic quality.^40,41^ The criteria contain 16 key components to evaluate the quality of radiomics studies and specify that large-scale data sharing is essential for the validation and full potential in radiomics. Hence, well-designed and multicenter investigation is needed to prove the clinical significance of radiomics and ultimately contribute to treatment decision-making and prognostic prediction that could be promisingly incorporated into clinical routine in the future.^40^

### Limitations

There are several limitations to this study. First, the tumor segmentation was manually contoured by different radiologists and was considered time-consuming and labor intensive. Automated tumor segmentation using machine learning or deep learning algorithms could address the issue efficiently; however, precise autosegmentation without manual edits remains challenging.^42^ A previous study reported that semiautomated segmentation on CT images improved the repeatability of radiomic features.^43^ In a multicenter study, manual and semi-automated segmentations may introduce considerable observer bias; however, we determined that manual tumor segmentation was reliable and robust by demonstrating excellent intraobserver and interobserver consistencies. Second, clinical markers, such as cancer antigen 125, were not incorporated into the model building. Recent studies demonstrated the potential value of combined radiomic features and clinical markers in predicting lymph node or extrapelvic metastasis in ovarian cancer.^35,37^ Additional radiomics studies that incorporate clinical markers will be of interest to investigate the clinical significance in predicting treatment response and prognosis of EOC. Third, this was a retrospective, multicenter study, which would limit the level of evidence in radiomics. Although most current radiomics investigations are retrospective, well-designed prospective studies will be expected to mature radiomics as a discipline in the future.

## Conclusions

This multicenter diagnostic study assessed the utility of CT radiomics in discriminating histologic subtypes of EOC. The proposed LR model of CT radiomic features selected by the voted LASSO method demonstrated excellent diagnostic performance in differentiating HGSC and non-HGSC.

## Notes

Supplement. **eTable.** CT Scanners and Parameters of 4 Centres **eFigure.** Workflow Diagram of Radiomics in EOC **eAppendix.** Definitions of the Selected Radiomic Features
