# Supplementary material for: Computed Tomographic Radiomics in Differentiating Histologic Subtypes of Epithelial Ovarian Carcinoma
Source: JAMA Netw Open. 2022 Dec 5;5(12):e2245141. doi: 10.1001/jamanetworkopen.2022.45141 (PMC9855300; doi:10.1001/jamanetworkopen.2022.45141)
Supplement: Supplement. — eTable. CT Scanners and Parameters of 4 Centres eFigure. Workflow Diagram of Radiomics in EOC eAppendix. Definitions of the Selected Radiomic Features [file jamanetwopen-e2245141-s001.pdf]

Supplementary Online Content

Wang M, Perucho JAU, Hu Y, et al. Computed tomographic radiomics in differentiating histologic subtypes of epithelial ovarian carcinoma. *JAMA Netw Open*. 2022;5(12):e2245141. doi:10.1001/jamanetworkopen.2022.45141

eTable. CT Scanners and Parameters of 4 Centres

eFigure. Workflow Diagram of Radiomics in EOC

eAppendix. Definitions of the Selected Radiomic Features

This supplementary material has been provided by the authors to give readers additional information about their work.

eTable. CT Scanners and Parameters of 4 Centres

Parameters	Centre A	Centre B	Centre C	Centre D
CT scanners	Toshiba Aquilion	GE LightSpeed VCT	Philips IQon Spectral	Siemens Somatom Force
	GE Discovery CT750 HD	Siemens Somatom Definition	Canon Aquilion Prime	Siemens Somatom Definition
	Philips iCT 256	Toshiba Aquilion Prime		GE Discovery CT750 HD
	Siemens Somatom Force			Toshiba Aquilion
Tube current (mA)	200-250	120-200	143-250	70-294
Tube voltage (kV)	120	120	120	70-130
Slice thickness (mm)	1.0	1.25	1.0	5.0
Pixel spacing	0.578-1.083	0.527-0.793	0.609-0.797	0.563-0.891

© 2022 Wang M et al. *JAMA Network Open.*

eFigure. Workflow Diagram of Radiomics in EOC

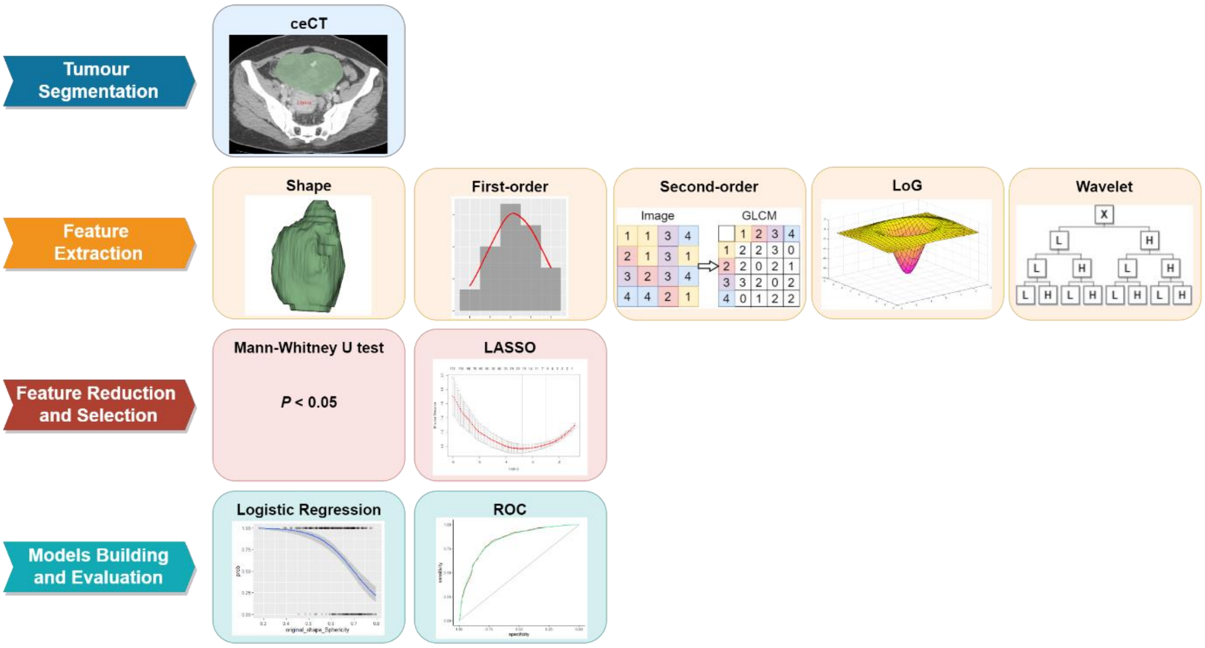

Abbreviation: ceCT, contrast-enhanced CT; LoG, Laplacian of Gaussian; LASSO, least absolute shrinkage and selection operator**.**

© 2022 Wang M et al. *JAMA Network Open.*

eAppendix. Definitions of the Selected Radiomic Features[27]

1. original_shape_Sphericity: original feature, shape class, a measure of the roundness of the shape of the tumour region relative to a sphere.
2. original_shape_MinorAxisLength: original feature, shape class, the second-largest axis length of the ROI-enclosing ellipsoid.
3. original_firstorder_90Percentile: original feature, first-order class, the 90th percentile of gray level intensity.
4. log1_glcm_Imc2: LoG transform (sigma=1), GLCM class, Informational Measure of Correlation 2 [assesses the correlation between the probability distributions of i and j (quantifying the complexity of the texture)].
5. log5_glszm_SmallAreaEmphasis: LoG transform (sigma=5), GLSZM class, the distribution of small size zones, with a greater value indicative of more smaller size zones and more fine textures.
6. log3_glcm_MaximumProbability: LoG transform (sigma=3), GLCM class, the occurrences of the most predominant pair of neighbouring intensity values.
7. wavelet_HHL_glszm_ZonePercentage: wavelet transform (high-high-low decomposition), GLSZM class, the coarseness of the texture by taking the ratio of number of zones and number of voxels.
8. wavelet_LLH_glszm_SizeZoneNonUniformity: wavelet transform (low-low-high decomposition), GLSZM class, the variability of size zone volumes in the image, with a lower value indicating more homogeneity in size zone volumes.
9. original_shape_Maximum2DDiameterSlice: original feature, shape class, the largest pairwise Euclidean distance between tumour surface mesh vertices in the row-column (generally the axial) plane.
10. log3_glszm_SmallAreaEmphasis: LoG transform (sigma=3), GLSZM class, the distribution of small size zones, with a greater value indicative of more smaller size zones and more fine textures.
11. log5_glrlm_LongRunHighGrayLevelEmphasis: LoG transform (sigma=5), GLRLM class, the joint distribution of long run lengths with higher gray-level values.
12. log1_glszm_SizeZoneNonUniformityNormalized: LoG transform (sigma=1), GLSZM class, the variability of size zone volumes throughout the image, with a lower value indicating more homogeneity among zone size volumes. This is the normalized version of the Size-Zone Non-Uniformity formula.
13. wavelet_LLL_gldm_DependenceVariance: wavelet transform (low-low-low decomposition), GLDM class, the variance in dependence size.
14. log1_firstorder_90Percentile: LoG transform (sigma=1), first-order class, the 90th percentile of gray level intensity.
15. original_gldm_DependenceNonUniformityNormalized: original feature, GLDM class, the similarity of dependence throughout the image, with a lower value indicating more homogeneity among dependencies. This is the normalized version of the Dependence Non-Uniformity formula.
16. wavelet_LLL_firstorder_Mean: wavelet transform (low-low-low decomposition), first-order class, the average gray level intensity.
17. wavelet_HLL_gldm_DependenceVariance: wavelet transform (high-low-low decomposition), GLDM class, the variance in dependence size.
18. wavelet_HHL_firstorder_Skewness: wavelet transform (high-high-low decomposition), first-order class, the asymmetry of the distribution of values about the Mean value.
19. log3_glszm_GrayLevelNonUniformityNormalized: LoG transform (sigma=3), GLSZM class, the variability of gray-level intensity values in the image, with a lower value indicating a greater similarity in intensity values. This is the normalized version of the Gray Level Non-uniformity formula.
20. wavelet_HHL_gldm_DependenceVariance: wavelet transform (high-high-low decomposition), GLDM class, the variance in dependence size.

© 2022 Wang M et al. *JAMA Network Open.*
